# *QuickStats*: Distribution* of Hours per Day That Office-Based Primary Care and Specialist Care Physicians Spent Outside Normal Office Hours Documenting Clinical Care in Their Medical Record System† — United States, 2019

**DOI:** 10.15585/mmwr.mm7050a4

**Published:** 2021-12-17

**Figure:**
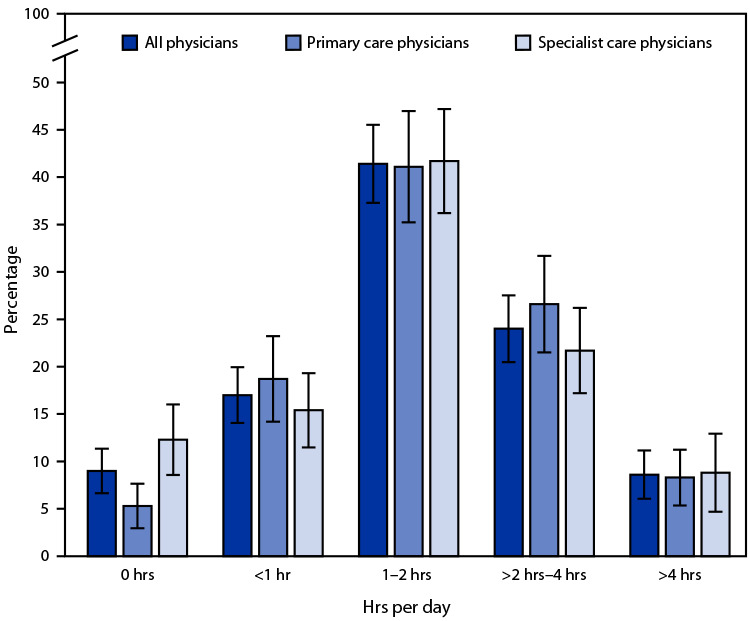
In 2019, 91.0% of office-based physicians spent time outside normal office hours documenting clinical care: 17.0% spent <1 hour, 41.4% spent 1–2 hours, 24.0% spent >2 hours–4 hours, and 8.6% spent >4 hours per day. The percentage of primary care physicians who spent no hours per day documenting clinical care (5.3%) was lower than the percentage of specialist care physicians (12.3%) who spent no hours per day documenting clinical care. In other time categories, there was no statistically significant difference between primary care and specialist care physicians.

**Source:** National Center for Health Statistics, National Electronic Health Records Survey, 2019. National Electronic Health Records Survey public use file national weighted estimates, 2019. https://www.cdc.gov/nchs/data/nehrs/2019NEHRS-PUF-weighted-estimates-508.pdf

